# Esophageal Cooling Device Versus Other Temperature Modulation Devices for Therapeutic Normothermia in Subarachnoid and Intracranial Hemorrhage

**DOI:** 10.1089/ther.2017.0033

**Published:** 2018-03-01

**Authors:** Imad Khan, Joseph Haymore, Brittany Barnaba, Michael Armahizer, Christopher Melinosky, Mary Ann Bautista, Brigid Blaber, Wan-Tsu Chang, Gunjan Parikh, Melissa Motta, Neeraj Badjatia

**Affiliations:** ^1^ Division of Neurocritical Care, Department of Neurology, University of Rochester School of Medicine and Dentistry, Rochester, New York.; ^2^ Department of Organizational Systems and Adult Health, University of Maryland School of Nursing, Baltimore, Maryland.; ^3^ Advanced Practice Provider Service, Department of Nursing, University of Maryland Medical Center, Baltimore, Maryland.; ^4^ Department of Nursing, University of Maryland Medical Center, Baltimore, Maryland.; ^5^ Department of Pharmacy, University of Maryland Medical Center, Baltimore, Maryland.; ^6^ Section of Neurocritical Care, Program in Trauma, Department of Neurology, University of Maryland School of Medicine, Baltimore, Maryland.

**Keywords:** esophageal cooling device, normothermia, targeted temperature management, subarachnoid hemorrhage, intracerebral hemorrhage

## Abstract

Achieving and maintaining normothermia (NT) after subarachnoid hemorrhage (SAH) or intracerebral hemorrhage (ICH) often require temperature modulating devices (TMD). Shivering is a common adverse effect of TMDs that can lead to further costs and complications. We evaluated an esophageal TMD, the EnsoETM (Attune Medical, Chicago, IL), to compare NT performance, shiver burden, and cost of shivering interventions with existing TMDs. Patients with SAH or ICH and refractory fever were treated with the EnsoETM. Patient demographics, temperature data, shiver severity, and amounts and costs of medications used for shiver management were prospectively collected. Controls who received other TMDs were matched for age, gender, and body surface area to EnsoETM recipients, and similar retrospective data were collected. All patients were mechanically ventilated. Fever burden was calculated as areas of curves of time spent above 37.5°C or 38°C. Demographics, temperature data, and costs of EnsoETM recipients were compared with recipients of other TMDs. Eight EnsoETM recipients and 24 controls between October 2015 and November 2016 were analyzed. There were no differences between the two groups in demographics or patient characteristics. No difference was found in temperature at initiation (38.7°C vs. 38.5°C, *p* = 0.4) and fever burden above 38°C (−0.44°C × hours vs. −0.53°C × hours, *p* = 0.47). EnsoETM recipients showed a nonsignificant trend in taking longer to achieve NT than other TMDs (5.4 hours vs. 2.9 hours, *p* = 0.07). EnsoETM recipients required fewer shiver interventions than controls (14 vs. 30, *p* = 0.02). EnsoETM recipients incurred fewer daily costs than controls ($124.27 vs. $232.76, *p* = 0.001). The EnsoETM achieved and maintained NT in SAH and ICH patients and was associated with less shivering and lower pharmaceutical costs than other TMDs. Further studies in larger populations are needed to determine the EnsoETM's efficacy in comparison to other TMDs.

## Introduction

Fever, defined as core body temperature greater than 38.3°C, has deleterious effects on the brain in the setting of acute brain injury (Greer *et al.*, 2008; O'Grady *et al.*, 2008). First-line treatment includes antipyretic medications such as acetaminophen and nonsteroidal anti-inflammatory agents. A diverse array of temperature modulating devices (TMDs) such as cooling blankets, wraps, and intravascular catheters are used for patients with fever refractory to pharmacologic treatment (Badjatia, 2009; Helbok *et al.*, 2012; Barr *et al.*, 2013). However, patients who are treated with these TMDs commonly experience shivering, which increases metabolic demand and oxygen consumption in the brain (Badjatia *et al.*, 2007, 2008; Hata *et al.*, 2008). Combating shivering often requires analgosedation, which can affect the neurologic examination, prolong mechanical ventilation, and prolong intensive care unit (ICU) length of stay (Choi *et al.*, 2011).

The EnsoETM is an esophageal TMD that can perform targeted temperature management (TTM). A previous case series of three patients demonstrated its ability to induce and maintain hypothermia in cardiac arrest patients (Hegazy *et al.*, 2015). We investigated the ability of the EnsoETM to treat refractory fever after nontraumatic brain hemorrhage and hypothesized that it would achieve normothermia (NT) with less shivering than currently available surface or intravascular TMDs.

## Methods

### Case selection

Adult patients with aneurysmal subarachnoid hemorrhage (SAH) or spontaneous intracerebral hemorrhage (ICH) with refractory fever were prospectively identified between October 2015 and April 2016 to receive the EnsoETM to restore NT. Refractory fever was defined as a febrile episode (temperature ≥38.3°C) that remained >38°C at least 2 hours after the administration of acetaminophen 650 mg enterally. Additional inclusion criteria were endotracheal intubation and hemodynamic stability. Exclusion criteria were as follows: (1) anticipated extubation, surgery, or withdrawal of support within 24 hours; (2) anticipated TTM for ≤72 hours; (3) active or recent upper gastrointestinal bleeding; (4) history of esophageal varices; (5) history of oral, esophageal, or gastric surgery or cancer; (6) history of hiatal hernia; and (7) any contraindication to orogastric (OG) tube placement.

### Control selection

A control group consisting of patients admitted with SAH or ICH between December 2015 and November 2016 was matched to EnsoETM patients by age, gender, and body surface area. These variables were chosen because of their strong association with shivering during TTM (Badjatia *et al.*, 2007; Choi *et al.*, 2011; Lyden *et al.*, 2012). All patients in the control group had fever refractory to acetaminophen and underwent TTM with Stryker Rapr.Round/Medi-Therm system (Stryker, Kalamazoo, MI), Arctic Sun 5000 (Medivance/Bard, Louisville, CO), or the Zoll COOL LINE intravascular cooling catheter/ThermoGard XP system (Zoll, Chelmsford, MA). Retrospective chart review was conducted to record hourly temperature, Bedside Shivering Assessment Scale (BSAS) scores, and antishivering medication administration. As in the EnsoETM group, only patients who underwent TTM for ≥72 hours were included.

### Esophageal temperature modulation device

The EnsoETM (Attune Medical, Chicago, IL) is a new, FDA-approved TMD that provides conduction cooling via closed-loop system temperature-controlled water circulation. The device is a silicone tube with three lumens that is inserted orogastrically. Two outer lumens are used for water circulation in a closed-loop system, through which temperature-modulated water flows into one lumen and returns out the other and through a Stryker Medi-Therm III temperature control machine (Stryker). The Medi-Therm III algorithmically controls circulating water temperature with input from a rectal or bladder temperature probe. A third, central lumen is available for gastric access with three distal side ports in the stomach.

Attune Medical provided EnsoETM devices at no cost for demonstration purposes. No other financial or material support was provided to the investigators for the conduct of this study.

### Cooling

All selected patients had existing large bore oro- or nasogastric tubes (OG/NG) removed before EnsoETM placement. Small-bore postpyloric OG/NG tubes were allowed to remain in position. The EnsoETM was placed orogastrically using standard bedside technique. A continuous temperature probe was placed in either the bladder or rectum. The EnsoETM was connected to the Medi-Therm III temperature management system. For the first two patients, the Medi-Therm III was then set to AUTO, RAPID cooling mode with set point 37°C. All subsequent patients were initially set on MANUAL, set point 4°C. Once the patients' temperature reached <37.5°C, the Medi-Therm III was set to AUTO, RAPID, 36°C.

### Shivering management

All shiver managements, including standing prophylactic medications and titrated infusions, were based on a previously published protocol (Choi *et al.*, 2011). Standing prophylaxis consisted of buspirone 30 mg orogastrically every 8 hours, magnesium 4 g every 8 hours as needed to target serum levels of 3–4 mg/dL, and acetaminophen 1000 mg every 6 hours for all patients during TTM. All EnsoETM and control patients were pretreated with meperidine (50 mg) intravenously 15 minutes before the initiation of cooling. Surface counterwarming using a BAIR Hugger blanket (3M Corporation, St. Paul, MN) set at maximum temperature (43°C) was used with all control patients, but not with case patients. The BSAS was assessed on all patients every hour by nursing staff. For patients with shivering (BSAS score >1), fentanyl or dexmedetomidine infusion, and/or meperidine were administered. If the hourly BSAS remained ≥1 despite these measures, medication infusions were uptitrated. Propofol infusion was added if the hourly BSAS still remained ≥1 after maximizing fentanyl or dexmedetomidine infusions. Neuromuscular blockade or dantrolene was only used for refractory shivering after patients were deeply sedated.

### Data collection

For cases, prospective data were collected every 15 minutes during the initial 2 hours and then hourly until the EnsoETM was discontinued. In addition to baseline demographic data, we collected bladder or rectal temperature, BSAS scores, dosage, and volume of all continuous dosage of bolus antishiver medications (meperidine, fentanyl, propofol, dexmedetomidine, neuromuscular blockade). Each bolus of medication, initiation of an infusion, and uptitration of infusions were counted as an antishivering intervention. We additionally noted whether any EnsoETM patient developed gastric bleeding, enteral nutrition intolerance, emesis during the TTM period, or any other EnsoETM-associated complications.

### Statistical analysis

We used multiple controls per case (3:1 ratio) to increase both power and precision (Song and Chung, 2010). The final sample of cases (*n* = 8) and controls (*n* = 24) was assessed using a noncentrality parameter *t*-statistic using a power assumption of 80% (*β* = 0.2) and significance of 5% (*α* = 0.05). This analysis demonstrated adequate power was achieved with a total sample of 31 (8 cases and 23 controls).

Efficacy of temperature management with the EnsoETM was identified as the time taken to achieve NT and time spent above NT during TTM. Fever burden (time spent above NT range) was calculated for >37.5°C and >38.0°C × time in hours. Comparative analyses between EnsoETM and control groups were performed for demographic variables, time to NT, duration of NT, fever burden, and number of pharmacologic interventions used for shivering. We obtained wholesale pricing data for all antishivering medications and compared costs between EnsoETM and non-EnsoETM patients. Comparative analyses were performed using Student's *t*-test and chi-square test wherever appropriate. A backward Wald multivariable linear regression was performed to identify factors associated with mean daily cost for antishivering medications. Data were analyzed using SPSS software (Version 24.0; IBM Corp, Armonk, NY).

Informed consent was obtained from all individual participants included in the study. All procedures performed in studies involving human participants were in accordance with the ethical standards of the institutional and/or national research committee and with the Declaration of Helsinki 1964 and its later amendments or comparable ethical standards. All data collections were approved by the University of Maryland School of Medicine Institutional Review Board.

## Results

### Demographics

Ten patients in total received an EnsoETM. Two patients were excluded from analysis: one patient due to duration of NT less than 48 hours and one patient due to early removal of the device to secure gastric access. This resulted in a final sample of eight EnsoETM recipients for analysis. Matched controls (*n* = 24) who received TTM with the Arctic Sun, Stryker Rapr.Round, and/or Zoll intravascular cooling catheter were selected for analysis. No differences were noted in baseline characteristics between the two groups, including age, sex, body surface area, body mass index, Glasgow coma scale (GCS) at initiation of therapy, diagnosis, or presence of intraventricular hemorrhage (Table 1). No device-related adverse events were experienced in any recipients of the EnsoETM or other TMDs.

**Table 1.** Baseline Characteristics of Patients Undergoing Therapeutic Normothermia

	*EnsoETM (*n* = 8)*	*Controls (*n* = 24)*	p
Age (years)	52 ± 16	53 ± 13	0.8
Women	5 (63)	12 (50)	0.7
BSA, m^2^	1.95 ± 0.2	2.05 ± 0.3	0.4
BMI, kg/m^2^	30.8 ± 13	31.6 ± 8	0.8
GCS	8 (7–9)	8 (7–9)	0.4
Diagnosis			
Subarachnoid hemorrhage	2 (25)	16 (67)	0.1
Intracerebral hemorrhage	6 (75)	8 (33)	0.1
IVH present	4 (50)	13 (54)	1.0
Temperature modulating device^a^			
Gaymar		17	N/A
Arctic Sun		8	
Zoll		2	

All continuous variables shown as mean ± standard deviation. GCS shown as median ± standard deviation. All proportions shown as number (percentages).

^a^ Total number of temperature modulating devices is greater than sample size due to one patient requiring both Arctic Sun and Zoll and one patient requiring Gaymar and Zoll.

BMI, body mass index; GCS, Glasgow coma scale; IVH, intraventricular hemorrhage; BSA, body surface area.

### Temperature management

**FIG. 1.**
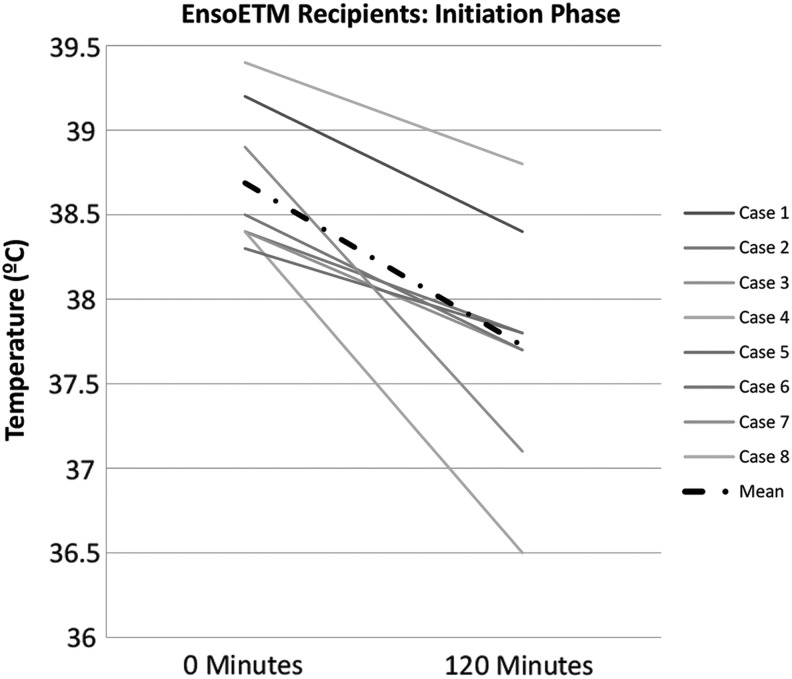
Initiation phase (initial 120 minutes) of induction of normothermia in EnsoETM recipients.

No difference in temperature at initiation of NT was noted between cases and controls (Table 2). The duration of TTM did not differ between recipients of the EnsoETM and other devices. EnsoETM recipients had a nonsignificant trend for longer time to target temperature of 37.5°C (Fig. 1 and Table 2) and higher fever burden than other TTM recipients. EnsoETM recipients received fewer median shiver interventions per day (3 [0–14] vs. 5 [0–21], *p* = 0.03) and over the total course of TTM (14 [5–35] vs. 30 [8–46], *p* = 0.02).

**Table 2.** Targeted Temperature Management Characteristics

	*EnsoETM (*n* = 8)*	*Controls (*n* = 24)*	p
Temperature at initiation (°C), mean ± SD	38.7 ± 0.4	38.5 ± 0.5	0.4
Time to target (hours), mean ± SD	5.4 ± 3.7	2.9 ± 3.2	0.07
Maintenance fever burden (°C × hours), mean ± SD			
Above 37.5	0.05 ± 0.25	−0.15 ± 0.28	0.09
Above 38	−0.44 ± 0.25	−0.53 ± 0.31	0.47
Shivering interventions per patient, median ± SD			
Total	14 (5–35)	30 (8–46)	0.02
Per day	3 (0–14)	5 (0–21)	0.03

SD, standard deviation.

### Antishivering medication costs

**FIG. 2.**
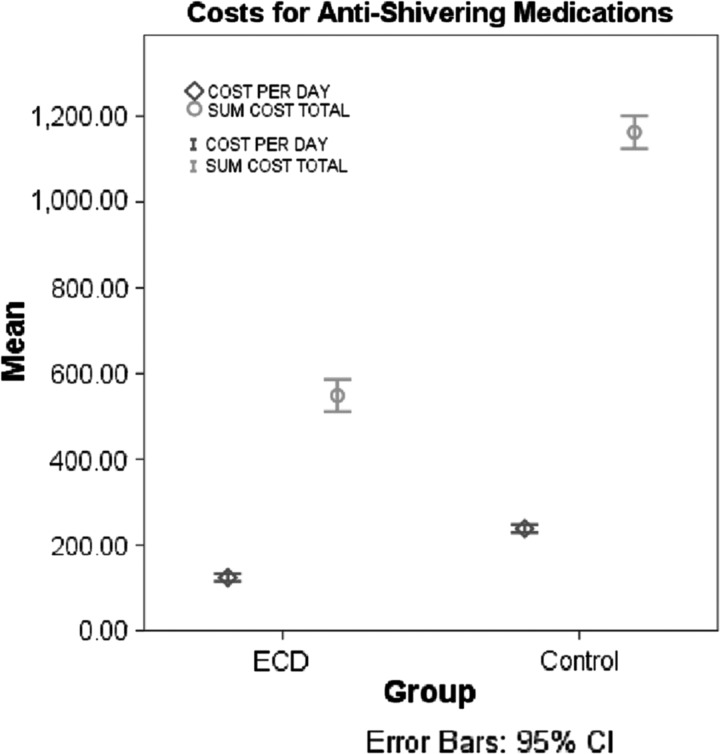
Average daily and total costs (in US$) per patient, grouped into EnsoETM recipients versus other TMD recipients (control). ECD, esophageal cooling device (EnsoETM); TMD, temperature modulating device.

EnsoETM recipients had lower daily costs for fentanyl, paralytics (rocuronium and cisatracurium), and dantrolene (Table 3). One patient required dantrolene and one patient required neuromuscular blockade, both in the control group. Comparing EnsoETM to non-EnsoETM patients, the mean daily cost ($124.27 ± 124.61 vs. $232.76 ± 253.31, *p* = 0.001) and total cost ($497.11 ± 543.18 vs. $1156.88 ± 1022.35, *p* = 0.03) for antishivering medications was less in EnsoETM patients (Fig. 2). In a multivariable linear regression model adjusting for sex, initial GCS, initial temperature, mean temperature during TTM, duration of TTM, and diagnosis, lower average daily antishivering medication cost was associated with older age (*β* = −6.4 ± 2.3, *p* = 0.01) and use of EnsoETM (*β* = −174.95 ± 89.8, *p* = 0.02) (*R*^2^ = 0.32).

**Table 3.** Mean Daily Cost per Patient for Shiver Suppression Pharmacologic Agents

	*EnsoETM (*n* = 8)*	*Controls (*n* = 24)*	p
Propofol	32.13 ± 27.89	83 ± 78.44	0.03
Fentanyl^a^	4.88 ± 5	12.76 ± 11.73	0.01
Dexmedetomidine	149.14 ± 84.18	197.84 ± 178.18	0.6
Meperidine	1.24 ± 0.98	2.18 ± 1.48	0.07
Paralytics^b^	0	48.46 ± 55.02	<0.0001
Dantrolene	0	383.16 ± 82.26	<0.0001

All costs reported in USD ± SD.

^a^ Includes continuous infusion and bolus doses.

^b^ Includes cisatracurium infusion and rocuronium boluses.

## Discussion

The EnsoETM system reduced temperature in patients with refractory fever and maintained NT for up to 120 hours. To our knowledge, this is the first reported use of this device to maintain TTM in ICH and SAH patients, as well as the first pharmacological cost analysis performed in its recipients. Previous case series describe the device's ability to induce and maintain therapeutic hypothermia in postcardiac arrest or coronary bypass patients, which represent populations with very different pathophysiology than cerebral hemorrhages (Hegazy *et al.*, 2015, 2017).

The EnsoETM was well tolerated in this intubated cohort of patients, and no adverse events were experienced with regard to placement or use of the device for TTM. This is consistent with a previous study that used the EnsoETM for therapeutic hypothermia in cardiac arrest (Markota *et al.*, 2016). No damage to the structural integrity of the device was ever noted after removal, and no patients experienced any symptoms related to esophageal damage, such as bleeding or strictures. Six of the eight EnsoETM recipients received gastric feeding via the device's central lumen, while the other two recipients were fed through existing nasogastric small-bore feeding tubes, which were left in place at the medical team's discretion. Lukewarm water was used to flush the EnsoETM's central lumen after crushed tablets were administered because one patient experienced transient clogging when cold water was used. One recipient of the EnsoETM was unable to receive gastric feeding due to a blockage of the central lumen, and the device was removed before NT could be achieved. This patient was not included in analysis. For all other recipients, no delays in enteral feeding or medication administration were noted, and no noticeable effect on TTM management was noted with the use of lukewarm flushes.

We found the requirement for medical interventions for shivering used by EnsoETM recipients to be significantly less than a matched cohort of patients undergoing NT with other TMDs. Our cost analysis found that this translated to a significant cost reduction related to the management of shivering with the EnsoETM. The thermoregulatory response of shivering is nearly universal during TTM and remains a substantial challenge for effective cooling. Shivering has been associated with an increase in metabolic demand, as previously described, which increases the resting energy expenditure and systemic rate of oxygen consumption (VO_2_) (Claessens-van Ooijen *et al.*, 2006; Badjatia *et al.*, 2008). It is possible that because of this, shivering has also been associated with tissue ischemia and increased morbidity, although further studies will need to be undertaken to prove this association (Ralley *et al.*, 1988; Oddo *et al.*, 2010).

All patients in this study had received our baseline antishivering regimen, which is designed to minimize the use of sedatives by first using IV magnesium, scheduled buspirone, and acetaminophen along with surface counterwarming. Previous literature notes that with this stepwise approach, the majority of patients required additional measures than this baseline regimen (e.g., opiates, dexmedetomidine) (Choi *et al.*, 2011). EnsoETM patients did not have counterwarming applied because no prior clinical or preclinical studies of this device used it (Kulstad *et al.*, 2012; Hegazy *et al.*, 2015, 2017; Williams *et al.*, 2016; Goury *et al.*, 2017). Even without counterwarming, EnsoETM recipients shivered less, required less medication, and did not require neuromuscular blockade. This stands in contrast with a previous case series describing the use of the EnsoETM in therapeutic hypothermia after cardiac arrest, which used neuromuscular blockade before initiation in all patients (Hegazy *et al.*, 2015).

Each medication used for shivering beyond our baseline regimen has significant adverse effects that must be considered in the care of the neurocritically ill patient (Paul and Paul, 2013). The use of meperidine, fentanyl, and propofol is associated with significant sedation, which can mask acute neurologic changes. These medications also have a cumulative effect over days of use and often lead to over sedation, which may prolong overall days of mechanical ventilation and length of stay in the ICU, and mask the clinical benefit of cooling in patients undergoing TTM (Badjatia, 2009; Barr *et al.*, 2013). Furthermore, given the frequent need to perform neurological assessment, pausing sedation hourly can result in a return of shivering and ensuing hypercapnia, hyperventilation, and overall hypermetabolism (Helbok *et al.*, 2012). Due to the minimized use of sedation for shiver control, we were often able to maintain EnsoETM patients in a more wakeful state and perform our neurological examinations with shorter pauses in sedation.

A cost savings was seen in the EnsoETM group due to the decreased requirement for pharmacologic antishiver interventions (Table 3). The most expensive medications by unit cost were dantrolene and cisatracurium, neither of which was used in EnsoETM recipients. Low costs as a barrier to utilization could justify more widespread use of NT in the neurologically injured patient population. However, further long-term studies will be needed to investigate whether the decrease in shiver burden and sedation usage results in shorter ICU stays, fewer ventilator days, and overall fewer costly complications, such as nosocomial infections.

We note several shortcomings of the EnsoETM in this study. The device was unable to be placed in one patient, which we hypothesize could have been due to a smaller, more acutely angled oropharynx. EnsoETM recipients took longer to achieve NT than recipients of other devices and had generally higher fever burdens. This may be explained by the limitations of the Medi-Therm III module, which has a maximal flow rate of 1.01 L/min (Gaymar Industries, 2010). In comparison, the Arctic Sun AS5000 and the Zoll ThermoGard XP modules have listed flow rates of 5 and 7 L/min, respectively (ZOLL Medical Corporation, 2012; Medivance, 2016). However, the EnsoETM is only designed to be used with the Medi-Therm III module.

There are several limitations to our study. Our primary goals were to discern whether achieving NT with an EnsoETM was possible and associated with less shivering, and thus, we selected to match controls across variables that are known to be associated with shivering. This resulted in a selection of nonconsecutive controls and may have introduced bias into our results. Another limitation is the adjustment in our practice made after the first two EnsoETM recipients, changing the cooling mode from “Automatic” to “Manual” set at 4°C until the patients reached target temperature. It is possible that this impacted the rate of cooling in the EnsoETM group. Although one could expect more shivering and medication use with this practice, the opposite was found to be true. A third limitation was the difference in number of SAH and ICH patients among the EnsoETM and other TMD groups. However, when factored into a multivariate logistic regression model for our primary endpoint, bleed type was not an independent predictor of shiver intervention requirements. Although we powered our study for our primary aim of shiver burden, we recognize that the overall sample size is small. The results from this study should be considered in this context. Our antishivering protocol is nurse driven and directed by the amount of shivering observed on an hourly basis as measured by the BSAS (Badjatia *et al.*, 2008). As a result, we believe investigators' biases did not influence medication utilization in both EnsoETM and non-EnsoETM patient groups.

## Conclusions

The EnsoETM is a novel temperature management system that induces and maintains a targeted temperature in intubated, febrile patients with intraparenchymal or SAHs and leads to fewer interventions for shivering. EnsoETM recipients had lower antishiver medication costs than recipients of other TMDs. Studies, including a broader patient population and longer durations of cooling, should be conducted to better understand how to optimally use this device among all other currently available TMDs.
